# Supplementary material for: Growth, Enzymatic, and Transcriptomic Analysis of xyr1 Deletion Reveals a Major Regulator of Plant Biomass-Degrading Enzymes in Trichoderma harzianum
Source: Biomolecules. 2024 Jan 24;14(2):148. doi: 10.3390/biom14020148 (PMC10887015; doi:10.3390/biom14020148)
Supplement: Supplementary file 1 [file biomolecules-14-00148-s001.zip › Figure S1.pdf]

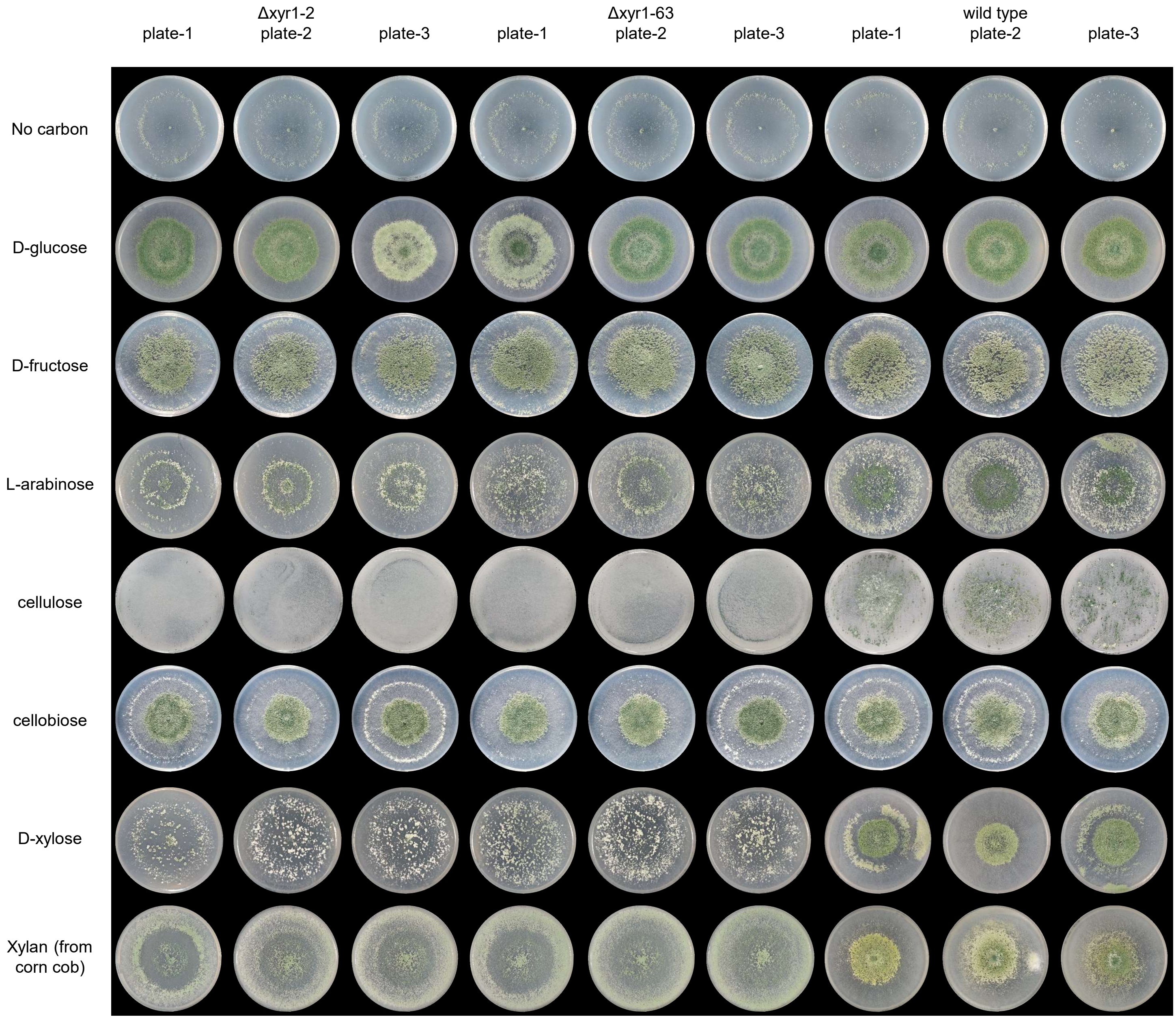

Figure S1. Images from 96 h from plate culture growth profiling analysis of *Trichoderma harzianum* Δxyr1 mutant.
