# Supplementary material for: Growth, Enzymatic, and Transcriptomic Analysis of xyr1 Deletion Reveals a Major Regulator of Plant Biomass-Degrading Enzymes in Trichoderma harzianum
Source: Biomolecules. 2024 Jan 24;14(2):148. doi: 10.3390/biom14020148 (PMC10887015; doi:10.3390/biom14020148)
Supplement: Supplementary file 1 [file biomolecules-14-00148-s001.zip › Figure S2.pdf]

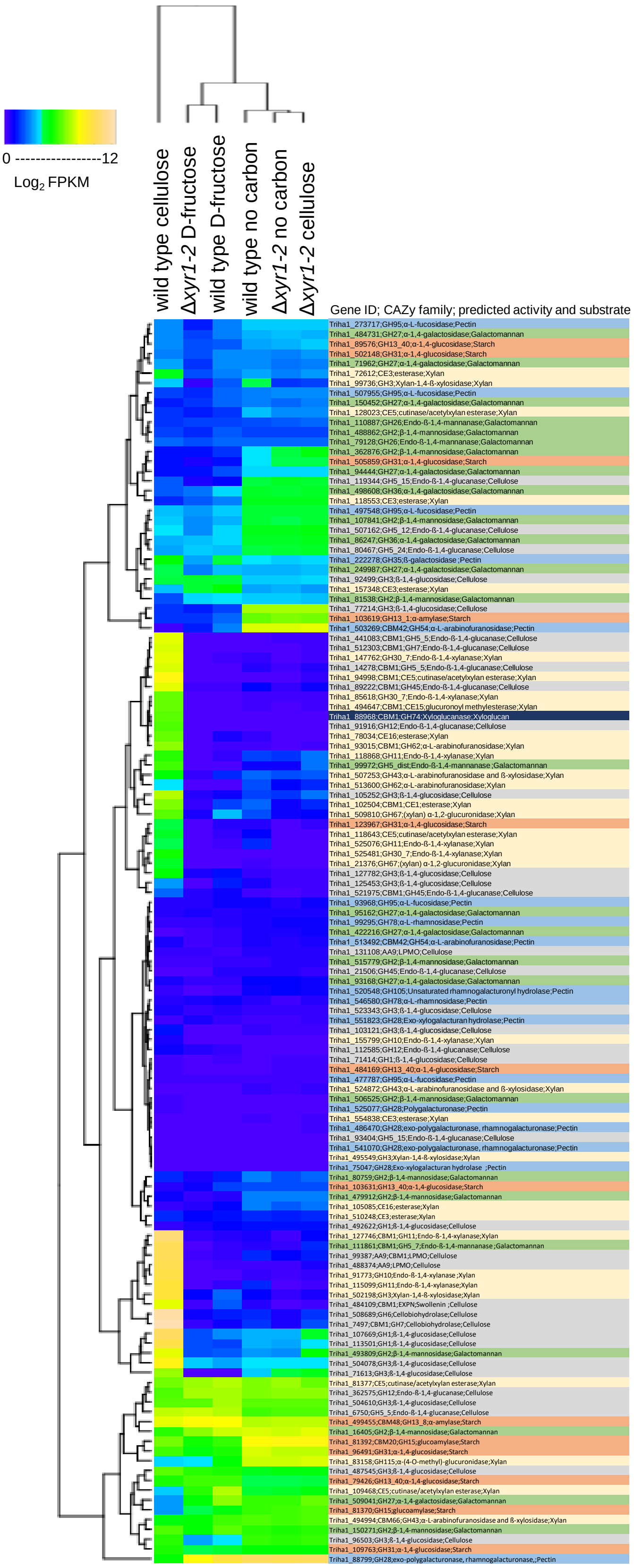

Figure S2. High-resolution image of the heatmap of the expression patterns of plant biomass-degrading (PBD) CAZy where the gene IDs and annotations for each of the rows of the heatmap are readable.
