# Supplementary material for: Growth, Enzymatic, and Transcriptomic Analysis of xyr1 Deletion Reveals a Major Regulator of Plant Biomass-Degrading Enzymes in Trichoderma harzianum
Source: Biomolecules. 2024 Jan 24;14(2):148. doi: 10.3390/biom14020148 (PMC10887015; doi:10.3390/biom14020148)
Supplement: Supplementary file 1 [file biomolecules-14-00148-s001.zip › Figure S3.pptx]

## Slide 1
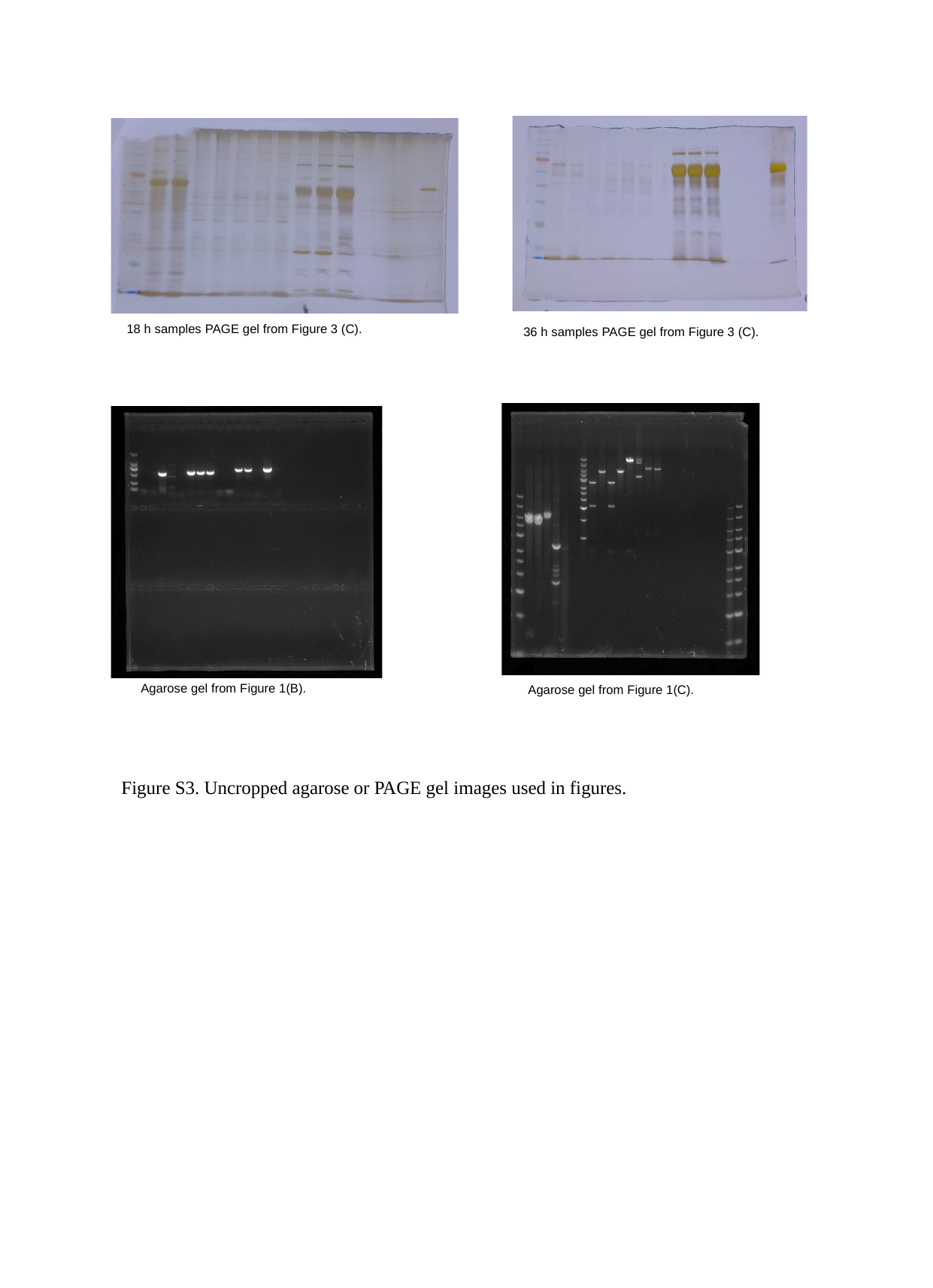

18 h samples PAGE gel from Figure 3 (C).
36 h samples PAGE gel from Figure 3 (C).
Agarose gel from Figure 1(B).
Agarose gel from Figure 1(C).
Figure S3. Uncropped agarose or PAGE gel images used in figures.
